# 

**Published:** 1894-02

**Authors:** 


### CONTENTS:

### OBITUARY.

Dr. Henry Snowden. - - - - - - - 433

### SELECTIONS.

Article I.—The Foil for Filling Teeth. By Dr. H. L. Ambler. - 439
" II.—The Treatment of Teeth During Pregnancy. By G. N. Skipp, L. D. S. - - - - - 450
" III.—Amalgam. By E. G. Clark, D. D. S. - - - 458
" IV.—Vaccination Ascribed as the Cause of a Death from Lockjaw. - - - - - - - 463
" V.—Prosthetics. Reported by Mrs. J. M. Walker. - 467
" VI.—The Teeth and Hair. By Dr. S. H. Guilford. - 471

### EDITORIAL, Etc.

The Maryland Dental Law. - - - - - - 475

### MONTHLY SUMMARY.

A Splendid Operation. - - - - - - - 479

Dental Dots Distilled. - - - - - - - 479

Remarkable Trepanning. - - - - - - - 480

A Plan for Destroying Pulps. - - - - - - 480

